# Cellulose accessibility limits the effectiveness of minimum cellulase loading on the efficient hydrolysis of pretreated lignocellulosic substrates

**DOI:** 10.1186/1754-6834-4-3

**Published:** 2011-02-10

**Authors:** Valdeir Arantes, Jack N Saddler

**Affiliations:** 1 Forestry Products Biotechnology/Bioenergy Group, Faculty of Forestry, University of British Columbia, 2424 Main Mall, Vancouver BC, V6T 1Z4, Canada

## Abstract

A range of lignocellulosic feedstocks (including agricultural, softwood and hardwood substrates) were pretreated with either sulfur dioxide-catalyzed steam or an ethanol organosolv procedure to try to establish a reliable assessment of the factors governing the minimum protein loading that could be used to achieve efficient hydrolysis. A statistical design approach was first used to define what might constitute the minimum protein loading (cellulases and β-glucosidase) that could be used to achieve efficient saccharification (defined as at least 70% glucan conversion) of the pretreated substrates after 72 hours of hydrolysis. The likely substrate factors that limit cellulose availability/accessibility were assessed, and then compared with the optimized minimum amounts of protein used to obtain effective hydrolysis. The optimized minimum protein loadings to achieve efficient hydrolysis of seven pretreated substrates ranged between 18 and 63 mg protein per gram of glucan. Within the similarly pretreated group of lignocellulosic feedstocks, the agricultural residues (corn stover and corn fiber) required significantly lower protein loadings to achieve efficient hydrolysis than did the pretreated woody biomass (poplar, douglas fir and lodgepole pine). Regardless of the substantial differences in the source, structure and chemical composition of the feedstocks, and the difference in the pretreatment technology used, the protein loading required to achieve efficient hydrolysis of lignocellulosic substrates was strongly dependent on the accessibility of the cellulosic component of each of the substrates. We found that cellulose-rich substrates with highly accessible cellulose, as assessed by the Simons' stain method, required a lower protein loading per gram of glucan to obtain efficient hydrolysis compared with substrates containing less accessible cellulose. These results suggest that the rate-limiting step during hydrolysis is not the catalytic cleavage of the cellulose chains *per se*, but rather the limited accessibility of the enzymes to the cellulose chains due to the physical structure of the cellulosic substrate.

## Background

Bioethanol derived from the bioconversion of lignocellulosic feedstocks continues to attract global interest as a potentially environmentally compatible alternative to current petroleum-based transportation fuels. However, considerable technical improvements are still needed before efficient and economically feasible lignocellulosic biomass-based bioethanol processes can be commercialized. One of the major limitations of this process is the consistently high cost of the enzymes involved in the conversion of the cellulose component into fermentable sugars [1]. This is primarily due to the comparatively high (compared with amylase loadings required for starch hydrolysis) protein loadings commonly required to overcome the substrate features and enzyme-related factors limiting effective cellulose hydrolysis [2]. Achieving rapid and complete enzymatic hydrolysis of lignocellulosic biomass at low protein loadings continues to be a major technical challenge in the commercialization of cellulose-based processes converting biomass to ethanol.

**Figure 1:**
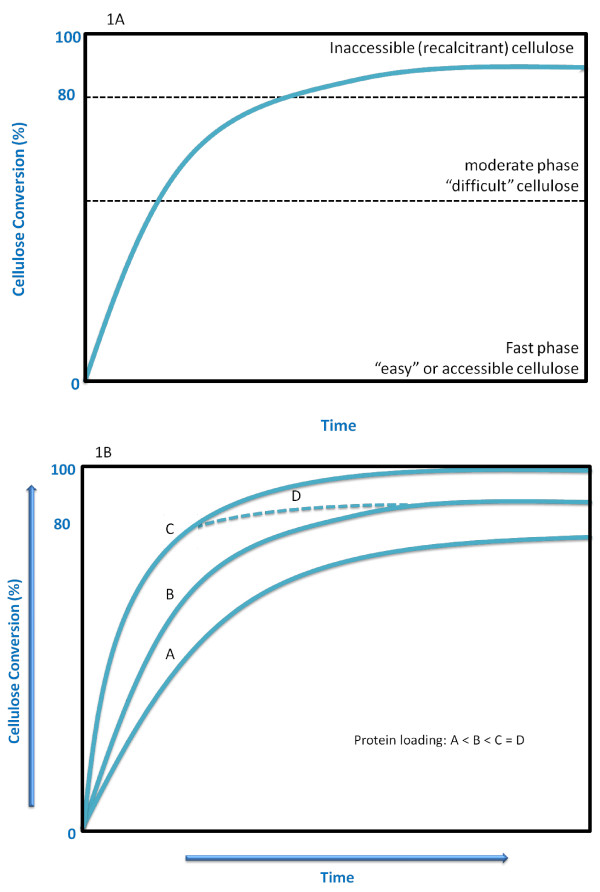
**Typical time course of (A) the enzymatic hydrolysis of cellulose; (B) cellulose hydrolysis with increasing protein loadings**.

In a typical batch enzyme-based process, cellulose conversion-time experiments are characterized by a three-phase curve (Figure 1A). This usually starts with the rapid adsorption of the cellulases onto the readily accessible cellulose, followed by an initial, fast rate of hydrolysis. However, the reaction quickly reaches an intermediate phase, characterized by a moderate hydrolysis reaction rate when about 50-70% of the original substrate has been hydrolyzed. Thereafter, a very slow phase is characterized by a steady decrease of the reaction rate, which results in only a slight increase in the conversion of the remaining (the so-called 'inaccessible' or recalcitrant) cellulose. Typically, extended hydrolysis times and/or high protein loadings are required to achieve a near-complete conversion of cellulose (Figure 1B). In some cases, depending on the nature of the substrate and the pretreatment method used, even at very high protein loadings of the commercially available cellulase mixtures (Figure 1B, curve D) and extensive hydrolysis times, complete cellulose hydrolysis cannot be achieved (Figure 1B) [3,4].

Previous technoeconomic modeling has shown that the long hydrolysis time associated with achieving complete cellulose saccharification adds significantly to the operating costs of the enzymatic hydrolysis step and, consequently, to those of the overall biomass to ethanol bioconversion process [5]. Recently, Shen and Agblevor [6] also studied the effect of hydrolysis time and enzyme loading on the hydrolysis of mixtures of cotton gin waste and recycled paper sludge with the aim of maximizing profit. This work indicated that the use of higher enzyme loadings to achieve >90% cellulose hydrolysis levels was difficult to justify because of the increased enzyme costs. In the work we describe here, we set a target of achieving at least 70% glucan hydrolysis of a range of lignocellulosic substrates, using the lowest possible enzyme loading.

Various substrate- and enzyme-related factors have been suggested to explain the slowdown in the rate of hydrolysis and, in many cases, the incomplete hydrolysis of cellulosic materials. Although there is still considerable debate about the contribution of each of these factors, it has been suggested that the accessible surface area of the cellulose is one of the most important factors influencing the rate and extent of enzymatic hydrolysis of lignocellulosic substrates [7-9]. This is not surprising, as the enzymatic hydrolysis of cellulose is a surface-dominated phenomenon, and direct physical contact between the cellulase enzymes and substrate must occur.

One of the major barriers faced by cellulase enzymes during lignocellulose hydrolysis is their limited access to much of the cellulose, which is buried within the highly ordered and tightly packed fibrillar architecture of the cellulose microfibrils [10]. Cellulosic materials are typically not smooth but rather heterogeneous porous substrates, and their available surface area can generally be divided into exterior and interior surfaces. The latter can consist of internal pores, fissures and micro-cracks, which typically arise from 'discontinuities' of the molecular packing built into the cellulose at the time the solid substrate is generated [11], or surface openings/internal slits, voids or spaces created by the removal of non-cellulosic cell wall components during pretreatment [12-14]. The external surface area of cellulosic-rich materials is largely determined by the individual overall fiber dimensions [15].

Earlier work by Grethlein [16] showed a linear correlation between the initial hydrolysis rate of pretreated biomass and the pore size accessible to a molecule with a diameter of 5.1 nm, which is about the diameter of a 'representative' cellulase. More recent work by Thygesen *et al*. [17], using fluorescent-labeled enzymes combined with confocal fluorescence microscopy, showed that cellulases were able to penetrate into the porous regions of the cellulose before any significant cellulose depolymerization was observed. Indeed, it has been suggested that the enzymatic hydrolysis of cellulose could occur both on the external surface by a sequential 'shaving' or 'planing' of the cellulose fibrils, or by key components of the cellulase mixture entering pores/fissures large enough to accommodate enzymes and then initiating the actual cellulose depolymerization process after a swelling action to increase substrate availability [10]. In either case, the cellulose topology/porosity can be expected to be an important factor that would influence the amount of protein adsorbed onto the substrate.

Although previous studies have highlighted the importance of cellulose accessibility during enzymatic hydrolysis [16-20], the majority of the studies have employed only a small number of samples and, in many cases, made use of a highly digestible 'model' or pure cellulosic substrates, which are not really indicative of how the realistic, natural heterogeneous, lignocellulosic feedstocks might behave. At the same time, the relationship between the substrate surface area and cellulose digestibility is sometimes contradictory and in many cases inconclusive. This is also, at least in part, probably due to the dependency of the accessible cellulose surface area on the nature of the substrate (for example, its source, pretreatment and storage) and the enzyme preparation used (complexity, type, composition, concentration), and on difference in the methods employed to assess changes. It is recognized that, some methods used to measure the available surface area of cellulosic materials are not particularly accurate (for example, water retention value, mercury porosimetry), and others involve drying the samples (for example, nitrogen adsorption technique). In the latter case, the pores of the wet cellulose fibers have been shown to shrink successively as the moisture content is decreased (an irreversible phenomenon termed 'hornification' [21]), resulting in smaller pore sizes and narrowed pore size distribution [22], which make the material less susceptible to enzymatic hydrolysis [20]. In addition to the difficulties experienced in measuring substrate changes occurring during hydrolysis, comparatively high enzyme dosages have been employed in many of these past studies, possibly masking any differences that might have been observed in the substrate characteristics. Thus, the influence that the specific surface area of the cellulose might have on our ability to achieve fast and complete enzymatic hydrolysis of pretreated lignocellulosic feedstocks at low protein loadings remains ambiguous. Therefore, further work, using a broad range of lignocellulosic substrates, moderate protein loadings, and substrate characterization methods that do not require sample drying, is warranted.

In the present study, a range of lignocellulosic feedstocks (including agricultural, softwood and hardwood substrates) were subjected to sulfur dioxide (SO_2_)-catalyzed steam and ethanol organosolv (EO) pretreatment at previously determined conditions [23-27] that were deemed optimal for both good hemicellulose recovery and subsequent hydrolysis of the cellulose-rich stream. These pretreated substrates were used to determine the key factors that might help achieve efficient hydrolysis at low enzyme loadings. With this goal in mind, a statistical design approach was first used to define what might constitute a minimum protein loading for the efficient hydrolysis of a range of pretreated substrates. In parallel, the substrate factors (that is, the external and internal surface area of cellulose-rich substrates) that might limit the accessibility of the cellulase complex to the cellulose and the maximum protein adsorption capacity were measured for each substrate, in an attempt to correlate cellulose accessibility with the minimum protein-loading requirement for efficient hydrolysis. We also evaluated the influence of increasing hydrolysis times and solids loadings on the minimum protein loading (cellulase and β-glucosidase) required to achieve efficient hydrolysis.

The aim of this work was that, by defining the minimum protein loading required to achieve efficient hydrolysis, it would help us to better understand which key factors limit the fast and near-complete hydrolysis of cellulosic substrates at moderate protein loadings. As indicated in the paper, the results provided us with some insights into how we could improve accessibility to the cellulose fibers/microfibrils, consequently improving the enzymatic digestibility of lignocellulosic materials.

## Materials and methods

### Enzyme preparations

Two commercial preparations (both Novozymes, Franklinton, NC, USA) - a cellulase cocktail (Celluclast 1.5 L; protein content 129.8 mg/mL) derived from *Trichoderma reesei *and a β-glucosidase preparation (Novozym 188; protein content 233 mg/mL) derived from *Aspergillus niger *- were used in the enzymatic hydrolysis experiments. Protein concentrations were determined using the modified ninhydrin method [28]. Bovine serum albumin was used as the protein standard.

### Lignocellulosic feedstocks and pretreatment technologies

Representatives of agricultural residues (corn stover and corn fiber), softwood (douglas fir and beetle-killed lodgepole pine) and hardwood (hybrid poplar) were used in this study.

Lignocellulosic feedstocks were pretreated by SO_2_-catalyzed steam and/or EO pretreatment as described previously [29]. Most of the pretreatments were performed at near-optimal pretreatment conditions (Table 1), which have previously been determined in our laboratories (steam-pretreated corn stover (SPCS) [25], corn fiber (SPCF) [23], douglas fir (SPDF) and lodgepole pine (SPLP) [27], and EO-pretreated lodgepole pine (OPLP) [26] and poplar (OPP) [24]) to obtain good overall carbohydrate recovery (that is, hemicelluloses and cellulose) while producing cellulose-rich substrates amenable to enzymatic hydrolysis. After pretreatment, all substrates (solid fractions) were thoroughly washed, filtered, and kept in refrigerated storage until they were used for analysis and hydrolysis.

**Table 1:** Pretreatment conditions and chemical composition of pretreated lignocellulosic substrates.

**Substrate**	**Pretreatment conditions**	**Composition of pretreated feedstocks**						**Abbreviation**
	
SO_2_-steam pretreatment^**a**^		**Ara**^**b**^	**Gal**^**c**^	**Glu**^**d**^	**Xyl**^**e**^	**Man**^**f**^	**AIL**^**g**^	
		
Corn stover	190°C, 5 minutes, 3% SO_2_	0.8	0.2	55.1	12.0	1.9	18.9	SPCS
Corn fiber	190°C, 5 minutes, 4% SO_2_	6.9	2.8	38.2	15.3	2.2	12.6	SPCS
Douglas fir	200°C, 5 minutes, 4% SO_2_	BDL^**g**^	BDL	50.6	0.4	1.0	47.0	SPDF
Lodgepole pine	200°C, 5 minutes, 4% SO2	BDL	BDL	52.4	0.6	1.0	45.9	SPLP
Ethanol-organosolv pretreatment								
Corn fiber	170°C, 30 minutes; 65% EtOH, 0.75% H_2_SO_4_	2.1	1.6	57.9	11.5	3.0	15.7	OPCF
Poplar	195°C, 40 minutes; 70% EtOH, 1.0% H_2_SO_4_	BDL	BDL	77.0	6.0	2.4	16.0	OPP
Lodgepole pine	170°C, 60 minutes; 65% EtOH, 1.1% H_2_SO_4_	0.1	0.1	74.8	1.6	1.8	17.3	OPLP

^a^Sulfur dioxide.

^**b**^Arabinan.

^**c**^Xylan.

^**d**^Glucan.

^**e**^Galactan.

^**f**^Mannan.

^**g**^Acid-insoluble lignin.

^**h**^Below detectable level.

### Chemical analysis of pretreated feedstocks

The chemical composition of the pretreated materials was determined according to a standard method (T222 om-88; Technical Association of the Pulp and Paper Industry), as previously described [30]. Monosaccharides were analyzed by high-performance liquid chromatography with fucose as the internal standard, as previously described [31]. All analyses were performed in triplicate. Carbohydrate and lignin contents are shown in Table 1.

### Defining minimum protein loadings for efficient hydrolysis

Optimization of minimum protein loadings required for efficient glucose release from a broad range of pretreated substrates was performed according to a central composite design in the form of a 2^4 ^full factorial design experiment with three central points. The dependent variable was glucan conversion, expressed as percentage, and the independent variables were the cellulase (Celluclast 1.5 L) and β-glucosidase (Novozym 188) loadings, hydrolysis time, and solids loading. The range and the levels of these variables are given in Table 2.

**Table 2:** Coded and actual levels of variables chosen for the statistical design of experiment.

Factors	Level	Pretreatment			
		
		**SO**_**2 **_**steam**^**a**^		Ethanol organosolv	
		
		**CS,**^**b **^**DF, **^**c **^**LP**^**d**^	**CF**^**e**^	CF	**LP, P**^**f**^
Solids loading, %	-1	2	2	2	2
	0	6	6	6	6
	1	10	10	10	10
Hydrolysis time, hours	-1	24	24	24	24
	0	48	48	48	48
	1	72	72	72	72
Cellulase,^g ^mg protein/g glucan	-1	25	5	13	20
	0	50	15	26	45
	1	75	25	39	70
β-glucosidase,^h ^mg protein/g glucan	-1	0	0	0	0
	0	15	15	10	10
	1	30	30	20	20

^a^SO = sulfur dioxide.

^b^CS = corn stover.

^c^DF = douglas fir.

^d^LP = lodgepole pine.

^e^CF = corn fiber.

^f^P = poplar.

^g^Celluclast 1.5L.

^h^Novozym 188.

To describe and predict the effect precisely and quantitatively, the hydrolysis data for each of the pretreated materials was fitted using a second-order polynomial model and Statistica software (version 6.0; Statsoft Inc., Tulsa, OK, USA).

### Enzymatic hydrolysis

Batch hydrolysis of pretreated substrates was carried out in sodium acetate buffer 50 mmol/L pH 4.8, supplemented with 0.02% w/v tetracycline and 0.015% w/v cyclohexamide, to prevent microbial contamination. The reaction mixtures (1 mL) were mechanically shaken in an orbital shaker incubator (Combi-D24 hybridization incubator, FINEPCR^®^, Yang-Chung, Seoul, Korea) at 50°C. The conditions for cellulase and β-glucosidase loadings, hydrolysis time, and solids loadings were determined according to the statistical design of experiments (Table 3). Glucose concentration was determined using a microscale enzymatic assay involving glucose oxidase and horseradish peroxidase as adapted by Berlin *et al*. [32]. Hydrolysis yields (%) of the pretreated substrates were calculated from the cellulose content as a percentage of the theoretically available cellulose in the pretreated substrate. Enzymatic digestibility of the pretreated materials refers to the enzymatic digestibility of cellulose only, unless otherwise stated.

**Table 3:** Matrix and results of a 2^4 ^full factorial design with centered face and three repetitions at the center point for steam- and organosolv-pretreated lignocellulosic substrates.

Run	Factors				Glucan to glucose, %						
	
Number	Time	Solids	**Cell.**^**a**^	**BG**^**b**^	**SPCF**^**c**^	**SPCS**^**d**^	**SPLP**^**e**^	**SPDF**^**f**^	**OPP**^**g**^	**OPCF**^**h**^	**OPLP**^**i**^
1	-1	-1	-1	-1	16	46	8	18	12	27	5
2	1	-1	-1	-1	20	59	22	28	12	48	22
3	-1	-1	-1	1	74	62	40	43	27	82	39
4	1	-1	-1	1	79	68	44	46	33	95	57
5	-1	-1	1	-1	31	57	37	44	39	48	27
6	1	-1	1	-1	29	71	60	42	58	82	85
7	-1	-1	1	1	53	73	90	64	88	98	78
8	1	-1	1	1	54	61	72	63	82	76	101
9	-1	1	-1	-1	5	32	16	15	13	14	12
10	1	1	-1	-1	16	42	25	24	26	28	24
11	-1	1	-1	1	52	41	33	33	34	43	34
12	1	1	-1	1	54	46	45	44	45	61	52
13	-1	1	1	-1	24	43	34	35	34	33	30
14	1	1	1	-1	37	53	52	52	56	62	57
15	-1	1	1	1	55	52	53	60	61	69	63
16	1	1	1	1	56	67	63	74	82	90	92
17	0	-1	0	0	78	73	72	70	65	98	84
18	0	1	0	0	59	53	47	52	63	70	68
19	0	0	-1	0	51	61	45	35	42	68	10
20	0	0	1	0	63	64	74	75	82	78	85
21	0	0	0	-1	31	55	40	42	37	43	38
22	0	0	0	1	61	65	71	68	63	91	79
23	-1	0	0	0	63	62	60	61	52	74	62
24	1	0	0	0	63	70	57	72	89	97	91
25	0	0	0	0	58	63	61	67	72	73	67
26	0	0	0	0	68	64	63	62	66	66	77
27	0	0	0	0	60	61	69	65	73	67	74

^a^Cellulase.

^b^BG = β-glucosidase.

^c^SPCF = steam-pretreated corn fiber.

^d^SPCS = steam-pretreated corn stover.

^e^SPLP = steam-pretreated lodgepole pine.

^f^SPDF = steam-pretreated douglas fir.

^g^OPP = organosolv-pretreated poplar.

^h^OPCF = organosolv-pretreated corn fiber.

^i^OPLP = organosolv-pretreated lodgepole pine.

### Available surface area

#### Protein adsorption

The maximum extent of protein (cellulase and β-glucosidase) adsorption was used as an indication of the surface area of a particular substrate available for protein binding. Protein adsorption isotherms were established by varying the amounts of protein (cellulase + β-glucosidase) added to the different pretreated substrates (2 mg/mL) in sodium acetate buffer (50 mmol/L, pH 4.8). The cellulase:β-glucosidase ratios were obtained by assessing the minimum protein loading required for efficient hydrolysis. Free protein was determined by measuring the amount of protein in the supernatant after incubation at 4°C and 150 rpm for 1 hour to reach equilibrium. Bound protein was calculated as the difference between free protein and the total protein initially added to the reaction medium. The protein content was determined using the ninhydrin assay [28]. The experimental data was fitted to the Langmuir adsorption isotherm using the following linearized form of the equation:

$$\text{1}/{\text{P}}_{\text{ads}}=\text{1}/{\text{P}}_{\text{max}}{\text{K}}_{\text{p}}+(\text{1}/{\text{P}}_{\text{max}})\text{P},$$

in which P is the concentration of unadsorbed protein (mg of protein/mL), P_ads _is the concentration of adsorbed protein (mg of protein/mg of substrate), P_max _is the maximal adsorbed protein (mg of protein/mg of substrate) and K_p _is the equilibrium constant (mL/mg of protein).

#### Fiber length

The external surface area of the cellulosic-rich substrates measured as the average fiber length of the pretreated substrates was determined using a high-resolution fiber quality analyzer (FQA) (LDA02; OpTest Equipment, Inc., Hawkesbury, ON, Canada) in accordance with the procedure described by Robertson *et al. *[33]. Briefly, a dilute suspension of fibers with a fiber frequency of 25 to 40 events per second was transported through a sheath flow cell where the fibers were oriented and positioned. The images of the fibers were detected by a built-in charge-coupled device (CCD) camera, and the length of the fibers was measured by circular polarized light. All samples were run in duplicate.

#### Simons' stain

Simons' stain (SS), a staining technique used in the pulp and paper industry to examine changes in the physical structure of pulp fibers under the microscope, and adapted for evaluating the pore structure (internal surface area) of cellulosic materials [34], was performed according to the modified procedure by Chandra *et al*. [35]. Pontamine fast orange 6RN (direct orange; DO) and Pontamine fast sky blue 6BX (direct blue; DB) dyes were used (Pylam Products Co. Inc., Garden City, NY, USA). Fractionation of DO was performed according to Esteghlalian *et al*. [20].

## Results and Discussion

It has been predicted that a diverse range of plant biomass will be needed to satisfy the projected demands for second-generation bioethanol [36]. As would be expected, different feedstocks (for example, agricultural residues vs. forest biomass) have significant qualitative and quantitative differences in their component and structural arrangements. Additionally, further differences such as composition/distribution and arrangement of components are introduced during the pretreatment step, and are heavily influenced by the pretreatment process employed. This variability is known to have a significant effect on the enzymatic hydrolysis step [37]. Therefore, our initial approach was to select a broad range of lignocellulosic feedstocks, including representatives of agricultural and forest biomass, and to pretreat these materials under conditions that allowed maximum hemicellulose recovery and good enzymatic hydrolysis of the cellulosic component. Subsequently, a statistical experimental design was used to define the minimum amounts of protein required for efficient hydrolysis of the pretreated substrates, in order to establish a reliable assessment of the factors governing the minimum protein loading required for efficient hydrolysis of each of the pretreated substrates.

The minimum protein loading for efficient hydrolysis was initially optimized before any correlation was made, to account for the possibility that previous predictions of the hydrolyzability of pretreated lignocellulosics based on either low or high protein levels might not be as meaningful or as accurate as predicted. For example, predictions based on low protein loadings might only include saccharification of the so-called 'easy/accessible' cellulose, and thus factors that control the digestibility of cellulose at high levels of conversion might not have been assessed. By contrast, experiments carried out at high protein loadings might, by saturating the substrate with enzymes, mask important factors limiting efficient hydrolysis.

The enzymatic digestibility of the seven pretreated samples using varying protein and solids loadings and hydrolysis times was assessed by monitoring the amount of glucose released (Table 3), and the effect of each of the variables and their interactions during hydrolysis was assessed by direct analysis of their statistical significance with a reliability of 95% (Table 4). This approach was chosen because the significance of the interactions between the variables would have been lost if the experiments were carried out using the classic methods of varying the level of one parameter at a time over a certain range, while holding constant the rest of the tested variables.

**Table 4:** Estimated effects (*P*-value at 95% confidence level) for glucan conversion during hydrolysis of various pretreated lignocellulosic substrates.

Factor	**SPCF**^**a**^	**SPCS**^**b**^	**SPLP**^**c**^	**SPDF**^**d**^	**OPP**^**e**^	**OPCF**^**f**^	**OPLP**^**g**^
Mean/Interc.	<0.0001	<0.0001	<0.0001	<0.0001	<0.0001	<0.0001	<0.0001
1							
Time (L ^h^)	0.1209	<0.0001**	0.0037*	0.0120*	0.0002**	<0.0001**	<0.0001**
Time (Q ^i^)	0.7302	0.4838	0.2711	0.6937	0.4510	0.3613	0.2255
2							
Consistency (L)	0.0056*	<0.0001**	0.0049*	0.1038	0.4281	<0.0001**	0.0295*
Consistency (Q)	0.2199	0.2965	0.4479	0.2823	0.4004	0.4919	0.2497
3							
Cellulase (L)	0.1387	<0.0001**	0.0000**	0.0000**	<0.0001**	<0.0001**	<0.0001**
Cellulase (Q)	0.0420*	0.1679	0.4215	0.0163*	0.1568	0.1228	0.0013*
4							
BG^j ^(L)	<0.0001**	<0.0001**	<0.0001**	<0.0001**	<0.0001**	<0.0001**	<0.0001**
BG (Q)	0.0001**	0.0145*	0.0783*	0.0171*	0.0005	0.0109*	0.0563*
1L by 2L	0.5554	0.7365	0.7954	0.3060	0.2724	0.6278	0.1400
1L by 3L	0.8592	0.2955	0.3451	0.8947	0.1263	0.2986	0.0122*
1L by 4L	0.5278	0.2785	0.2945	0.7946	0.7840	0.3451	0.8609
2L by 3L	0.0179*	0.8421	0.0087*	0.6421	0.0059*	0.9680	0.0537*
2L by 4L	0.1884	0.1729	0.0197*	0.6285	0.1901	0.0372*	0.2298
3L by 4L	0.0006*	0.2711	0.2314	0.7789	0.0293*	0.5689	0.1829

*R*^2^	0.9692	0.9692	0.9549	0.9529	0.9704	0.9538	0.9589

^a^SPCF = Steam-pretreated corn fiber

^b^SPCS = steam-pretreated corn stover.

^c^SPLP = steam-pretreated lodgepole pine.

^d^SPDF = steam-pretreated douglas fir.

^e^OPP = organosolv-pretreated poplar.

^f^OPCF = organosolv-pretreated corn fiber.

^g^OPLP = organosolv-pretreated lodgepole pine.

^h^L = linear.

^i^Q = quadratic.

^j^BG = β-glucosidase.

*Significant model terms; ** highly significant model terms.

**Figure 2:**
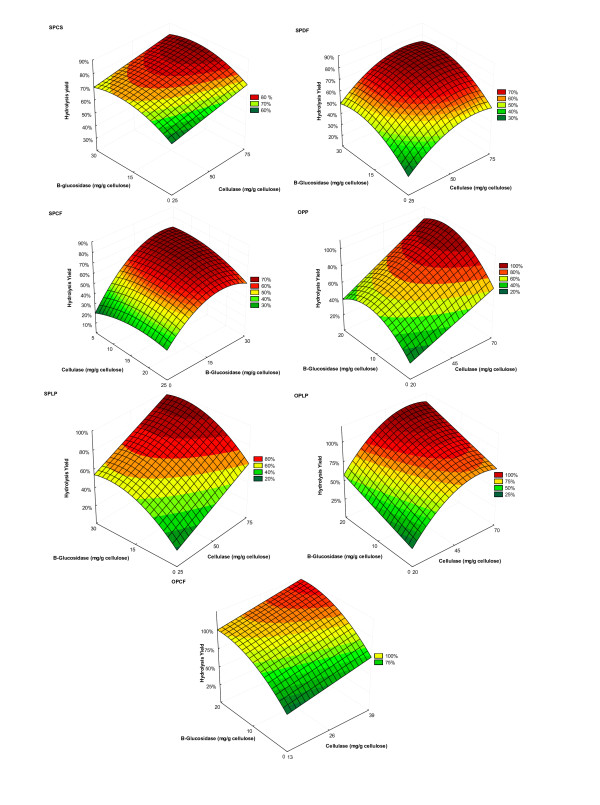
**Response surface fitted to the experimental data corresponding to the hydrolysis of a broad range of pretreated substrates**. Hydrolysis times and solids loadings were kept constant at 72 hours and 2% (w/v), respectively.

Regression analyses (ANOVA) were carried out to obtain mathematical models (Table 5) that better describe the relation between the independent variables (cellulase loading, β-glucosidase loading, hydrolysis time, and solids loading) and the studied response (glucose released). To prepare the adjusted models and their surfaces (Figure 2), only terms found to be significant at *P *≤ 0.05, or values near to this, were included in the models. The validity of the models was evaluated as a function of their respective coefficients of determination (*R*^2^). The value of the correlation coefficient provides a measure of variability in the observed response values that can be explained by the experimental factors and their interactions (the closer the *R*^2 ^value to 1.0, the better the fit of the model to the experimental data). The models computed for the *R*^2 ^value ranged between 0.91 and 0.96 (Table 5), indicating that the models were appropriate and could be used for quantitative prediction of the minimum protein loadings (cellulase and β-glucosidase) required to attain efficient cellulose conversion, and for assessment of the effect of time and solids loading during hydrolysis.

**Table 5:** Predictive models describing the relationship between hydrolysis yields of various pretreated lignocellulosic substrates and the significant variables.

Substrate	**Model**^**a**^	***R***^**2**^
SPCF^**b**^	H = 0.1349 + 0.00085T - 0.0257S + 0.0214C - 0.0006C^2 ^+ 0.0412B - 0.00075B^2 ^+ 0.00096SC - 0.00043CB	0.9563
SPCS^**c**^	H = 0.4346 + 0.002T - 0.0229S + 0.0023C + 0.0131B - 0.00032B^2^	0.9406
SPLP^**d**^	H = -0.2426 + 0.0023T + 0.0234C + 0.0095C + 0.0327B - 0.0006B^2 ^- 0.0005CB - 0.0007SC	0.9163
SPDF^**e**^	H = -0.2953 + 0.0018T - 0.0065S + 0.0221C - 0.000171C^2 ^+ 0.02152B - 0.00047B^2^	0.9402
OPP^**f**^	H = -0.2409 + 0.0032T + 0.0207S + 0.0094C + 0.05006B - 0.00217B^2 ^- 0.0005C + 0.00015CB	0.9434
OPCF^**g**^	H = 0.1178 + 0.00438T - 0.0214S + 0.00876C + 0.0552B - 0.0014B^2 ^- 0.0012SB	0.9295
OPLP^**h**^	H = -0.5703 + 0.00166T + 0.061S + 0.0312C - 0.00028B^2 ^+ 0.0301B - 0.00064B^2 ^+ 0.00009TC - 0.00037SC	0.9294

^a^H = hydrolysis yield; C = cellulase loading, mg protein/g glucan; B = β-glucosidase loading, mg protein/g glucan; T = hydrolysis time, hours; S = solids loading, % w/v.

^b^SPCF = steam-pretreated corn fiber.

^c^SPCS = steam-pretreated corn stover.

^d^SPLP = steam-pretreated lodgepole pine.

^e^SPDP = steam-pretreated douglas fir.

^f^OPP = organosolv-pretreated poplar.

^g^OPCF = organosolv-pretreated corn fiber.

^h^OPLP = organosolv-pretreated lodgepole pine.

### Determining the minimum cellulase and β-glucosidase requirement for efficient hydrolysis

The commercial cellulase cocktail (Celluclast 1.5 L) derived from the filamentous fungus *T. reesei*, consists mainly of cellobiohydrolases and endoglucanases [38-40]. Owing to the low level of *in situ *β-glucosidase activity, this *T. reesei *cellulase system is commonly supplemented with an excess of β-glucosidase to avoid any end-product inhibition caused by the accumulation of cellobiose, which would mask the actual minimum cellulase requirement at higher levels. It has also been reported that synergism between cellulase enzymes decreases at high cellulase concentrations (around saturation levels) [41]. Thus, to avoid the use of excess protein and to take maximum advantage of the synergistic effect between the cellulases, the minimum β-glucosidase supplementation required for efficient hydrolysis of various pretreated lignocellulosic substrates was also determined.

In this work, we defined effective hydrolysis as at least 70% of the original cellulose in the pretreated lignocellulosic materials being hydrolyzed to glucose. With this percentage conversion as a target, a meaningful assessment of cellulose saccharification could be made before the typical, significant slowdown in hydrolysis rate took place (Figure 1A). As mentioned earlier [6], a recent economic assessment of the influence of protein loading on the maximum profit rate for ethanol production from biomass substrates suggested that the costs involved in achieving complete hydrolysis are prohibitive, and that fast but incomplete hydrolysis, leaving about 10-20% of the original substrate unhydrolyzed, might be a more effective strategy.

The effect of cellulase and β-glucosidase loading on cellulose saccharification yields for most pretreated substrates was significantly affected by cellulase and β-glucosidase loadings in the linear term, and less significantly affected by their interaction with each other, and by their interaction with solids loading and hydrolysis time (Table 4). Unexpectedly, the cellulase loading (linear term) was not significant for SPCF. The likely cause for this lack of significance is the heterogeneity of this pretreated substrate (observed visually) making accurate representative sampling unfeasible and resulting in the relatively high standard deviation observed with the hydrolysis yields (Table 3, runs 25-27). The other pretreated substrates were more homogeneous, exhibiting a mudlike consistency, and were thus easier to sample representatively.

The significance of the quadratic coefficients of cellulase loading for SPCF, SPDF and OPLPP, and of β-glucosidase loading for SPCS, SPCF, SPLP, SPDF, OPCF, OPLPP, indicate that cellulose saccharification yields increase with protein loading up to a certain level (Table 4). Beyond that, the entire variable has an inhibitory effect on cellulose conversion. It was apparent that, within the range of pretreated substrates and cellulase and β-glucosidase loadings used in the present study, hydrolysis yields were influenced more by high β-glucosidase than by high cellulase loadings.

The mathematical models (Table 5) obtained after regression of the results shown in Table 3 were used to quantitatively predict the minimum protein requirement for efficient hydrolysis (70% glucan conversion) (Table 6). Considering the difference in the degrees of hydrolyzability of the pretreated substrates (Table 3), the hydrolysis time was kept at 72 hours to ensure that cellulose conversion yields reached, or were near to, the 'plateau phase' of hydrolysis. Solids loadings were kept at 2% (w/v), in an attempt to generate data that could be further correlated with the protein adsorption data obtained from experiments that were also carried out at a 2% (w/v) solids loading. The influence of hydrolysis time and solids loading on the hydrolysis yields of the pretreated materials and on the minimum protein required to achieve efficient hydrolysis were next assessed.

**Table 6:** Minimum cellulase and β-glucosidase loadings required for efficient hydrolysis (70% glucan conversion) of a broad range of pretreated lignocellulosic substrates as predicted by the equations shown in Table (5) for 2% (w/v) solids loading and 72 hours.

Substrate	**Cell.**^**a**^	**BG**^**b**^	Cell./BG	Total
SPCF^**c**^	5	18	0.3	23
SPCS^**d**^	30	24	1.3	54
SPLP^**e**^	42	21	2.0	63
SPDF^**f**^	45	16	2.8	61
OPP^**g**^	38	10	3.8	48
OPCF^**h**^	14	4	3.5	18
OPLP^**i**^	32	11	2.9	43

^a^Cell = cellulase (Celluclast 1.5), mg protein/g glucan.

^b^BG = β-glucosidase (Novozym 188) mg protein/g glucan.

^c^SPCF = steam-pretreated corn fiber.

^d^SPCS = steam-pretreated corn stover.

^e^SPLP = steam-pretreated lodgepole pine.

^f^SPDF = steam-pretreated douglas fir.

^g^OPP = organosolv-pretreated poplar.

^h^OPCF = organosolv-pretreated corn fiber.

^i^OPLP = organosolv-pretreated lodgepole pine.

The reliability of the equations was also assessed by comparing the experimental values of the responses at the centre point conditions, an average of three independent experiments (Table 3, runs 25-27), with the values calculated using the equations shown in Table 5. The results (data not shown) indicated that the predicted values agreed well with the observed values for the hydrolysis yields (Table 6). All of the predicted minimum protein loadings fell within the range accurately predicted by the empirical models.

It was apparent that the minimum protein requirement ranged between 18 and 63 mg protein per gram of glucan. The minimum protein requirement increased as follows: OPCF < SPCF < OPLP < OPP < SPCS < SPDF < SPLP. It was observed that for the same feedstock (for example, corn fiber and lodgepole pine), EO pretreatment generally resulted in substrates that required less protein to achieve efficient hydrolysis than did steam pretreatment. Within the group of feedstocks pretreated by the same process, the pretreated agricultural residues (corn stover and corn fiber) required lower protein loading per gram of glucan to achieve high glucan conversion than did the pretreated forest biomass (poplar, douglas fir and lodgepole pine). This confirmed that the nature of the lignocellulosic feedstock plays an important role in determining the amount of protein required for effective hydrolysis. This was not unexpected, as the plant cell-wall architecture and molecular structure, which are the primary lignocellulosic factors contributing to biomass recalcitrance, are likely to be different in woody biomass and herbaceous plant-derived biomass. For instance, softwoods have a more rigid structure and a higher lignin content, and are therefore expected to display more resistance towards deconstruction (be more recalcitrant) than the less structurally recalcitrant biomass derived from herbaceous plants.

### Solids loading and hydrolysis time

**Figure 3:**
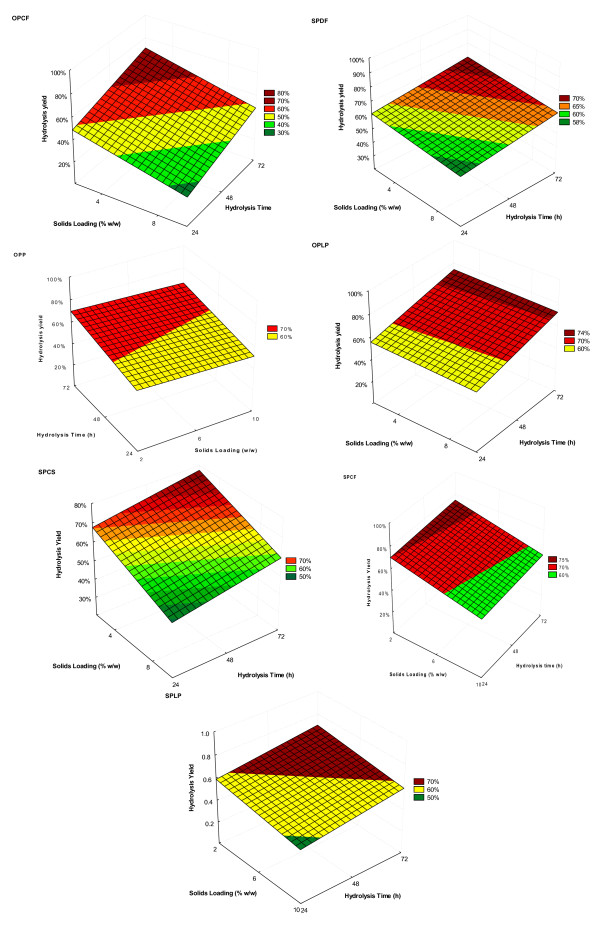
**Effect of hydrolysis time and solids loading on the minimum protein requirement for efficient hydrolysis of a variety of lignocellulosic substrates**. Cellulase and β-glucosidase were kept constant according to the protein level shown in Table 6.

To achieve efficient bioconversion of cellulose to ethanol, it is desirable that the hydrolyzate obtained after enzymatic hydrolysis contains a sufficiently high concentration of fermentable sugars to result in a high ethanol concentration. To obtain this high sugar concentration, hydrolysis should be carried out at high solids loading. Raising the solids loading in the enzymatic hydrolysis step is crucial to minimizing subsequent distillation costs, and it is also expected to decrease process cost by lowering the reactor size and minimizing water requirements [42,43]. It has been shown that the time required to achieve high conversion rates also contributes to the poor economics of the hydrolysis step [5]. Therefore, as well as determining the minimum protein loading required for efficient cellulose hydrolysis, the influence of time and solids loading on hydrolysis yields (Table 4) were also assessed for all of the pretreated substrates (Figure 3).

Regardless of the pretreatment process used, the effect of solids loading was highly significant for all of the pretreated agricultural residues (SPCF, SPCS and OPCF) (Table 4). This negative effect was probably due to the higher xylan content in these materials, which at high solids loading would be likely to result in the release of high concentrations of xylooligomers, which have been shown to inhibit the action of cellulases [44]. When the pretreated woods were assessed, the solids loading was only significant for the SPLP substrate. It was apparent that the interactive effect of solids loading with cellulase loading (2L by 3L) was more significant than the interactive effect with β-glucosidase (2L by 4L) (Table 4), but no correlation was observed between different feedstocks or pretreatments.

The linear effect of hydrolysis time was significant for all of the pretreated substrates, with the exception of the SPCF sample (Table 4). This lack of significance indicated that increasing the hydrolysis time from 24 h to 48 h or 72 hours does not necessarily result in statistically higher hydrolysis yields for the SPCF substrate, suggesting that the 'plateau phase' was reached within the first 24 hours of hydrolysis within the range of protein loadings used in this work. Previous results have shown that the SPCF substrate can be effectively hydrolyzed within 24 hours when moderate protein loadings are used [45].

The hydrolysis yields obtained with minimum protein loading for the steam pretreated wood substrates (SPDF and SPLP) did not seem to be affected by increasing the hydrolysis time from 24 hours to 72 hours, and the hydrolysis yields for the steam-pretreated agricultural residues (SPCF and SPCS) were only slightly affected (Figure 3). The greatest influence of hydrolysis time on the hydrolysis yields at minimum protein loading was observed with the EO pretreated samples (OPP, OPCF and OPLP) (Figure 3).

When the effect of solids loading on the hydrolysis efficiency of the pretreated materials at the optimized minimum protein loading was assessed (Figure 3), it was apparent that increasing the substrate concentration from 2% to 10% (w/v) decreased the hydrolysis yields for the SPCS, OPCF, SPCF and OPP substrates, whereas it had no effect on the yields for the SPDF, OPLP and SPLP substrates. The latter group of substrates had very low or undetectable levels of xylan, whereas the former group of samples had a relatively high xylan content. Again, this negative effect of solids loading on the hydrolysis yields was probably a result of inhibition of cellulase enzymes by high concentrations of xylooligomers at these higher substrate concentrations. Although the cellulase:β-glucosidase ratio was optimized for minimum protein loading at a 2% solids loading, it is possible that at higher substrate concentrations, cellooligomers might be produced. As cellooligomers inhibit cellulases as potently as do xylooligomers, this might also contribute to cellulase inhibition, thereby lowering hydrolysis yields at high solids loading, as a result of limited β-glucosidase levels.

### Protein adsorption

**Figure 4:**
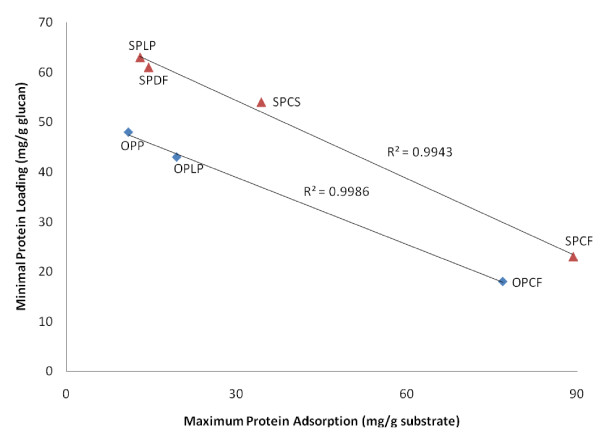
**Relationship between maximum protein adsorption capacity of a range of pretreated lignocellulosic biomass and the optimized minimum protein loading for efficient hydrolysis**.

The binding of cellulase enzymes onto insoluble and heterogeneous lignocellulosic biomass has been reported to have a strong role in governing the rates and yields of hydrolysis of cellulose [3,46], and also to be influenced by the available surface area of cellulose [46,47]. Therefore, the maximum cellulase adsorption was used as a parameter to measure the accessibility of the seven pretreated lignocellulosic substrates. Mixtures of cellulase and β-glucosidase over a range of concentrations were incubated with 2% (w/v) of pretreated material. The ratios of cellulase to β-glucosidase were based on the optimized minimum cellulase and β-glucosidase loadings required for efficient hydrolysis (Table 6). When the maximum amount of protein adsorbed onto the substrates was determined by fitting the experimental data to the Langmuir adsorption isotherm model, a good correlation (*R*^2 ^> 0.9790) was obtained. An assessment of the Langmuir adsorption isotherm revealed significant differences for protein adsorption onto the different pretreated lignocellulosic materials. The maximum adsorption capacity (P_max_) of proteins onto pretreated materials ranged from 11.0 to 89.3 mg/g substrate and increased as follows: steam-pretreated samples: SPLP < SPDF < SPCS < SPCF; then EO-pretreated samples: OPP < OPLP < OPCF (Figure 4).

When the optimized minimum protein loading for efficient hydrolysis was plotted against the accessibility of the substrate, determined as the maximum amount of protein that was adsorbed (P_max_), two distinct curves were observed (Figure 4). Each fitted curve corresponded to substrates pretreated by the same process. It appears that different pretreatment technologies have different effects on the adsorption of proteins onto the substrates, and that different feedstocks undergo similar modifications during the same pretreatment. The different protein adsorption patterns observed for the EO- and steam-pretreated samples may be, at least in part, a result of differences in the content and structure of the lignin, which is also known to bind proteins [48]. It has been reported that steam-pretreated substrates contain more binding sites on the lignin-particle surface due to the preservation of functional groups (phenolic hydroxyl and benzyl) and lignin branches during this type of pretreatment than do substrates produced by organosolv pretreatment, which is a delignifying process that decreases the total lignin content [49,50]. This suggests that protein adsorption patterns can be compared between different feedstocks pretreated by the same pretreatment technology, but not when pretreated by different pretreatment technologies.

The high correlation values, *R*^2 ^= 0.993 for the steam-pretreated and *R*^2 ^= 0.999 for EO-pretreated samples, indicate the strong dependency of the minimum protein loading required to achieve efficient hydrolysis on the maximum capacity (P_max_) of the substrates to bind protein.

For feedstocks pretreated by the same pretreatment technology, it was observed that the higher the capacity of the substrate to adsorb proteins, the lower the amount of protein required to attain efficient hydrolysis. This suggests that the more available the surface area of the cellulose-rich material for the proteins to bind to, the lower the protein-loading requirement for efficient cellulose saccharification. We next wanted to confirm the role that the available surface area of cellulose might have on the minimum protein loading required for efficient hydrolysis of lignocellulosic substrates.

### External and internal surface area versus minimum protein loading

It has been suggested that the cellulose surface area accessible to the cellulase enzymes is one of the most important factors determining the ease of hydrolysis of cellulosic materials, and it is also affected by several substrate characteristics. These features include distribution of particle size, pore volume, degree of crystallinity and degree of polymerization (DP) [9,35,37,46,51], among others. Although previous work has tried to correlate DP and crystallinity with enzymatic digestibility of cellulosic materials, using a comparison between the hydrolysis of a fully bleached eucalyptus Kraft pulp and that of SO_2_-catalyzed steam-pretreated eucalyptus chips, the substrate accessibility to the cellulases could not be readily predicted from the differences in their cellulose DP or crystallinity, but these substrate characteristics did indicate the likely mode of action of the enzymes [52]. From this and other work, it has been shown that the specific surface area of a mixture of particles is inversely proportional to the average diameter of the particles. Therefore, a smaller average particle size results in an increased surface area. Thus, it could be anticipated that a relationship between particle size and cellulose hydrolysis would occur [9].

**Figure 5:**
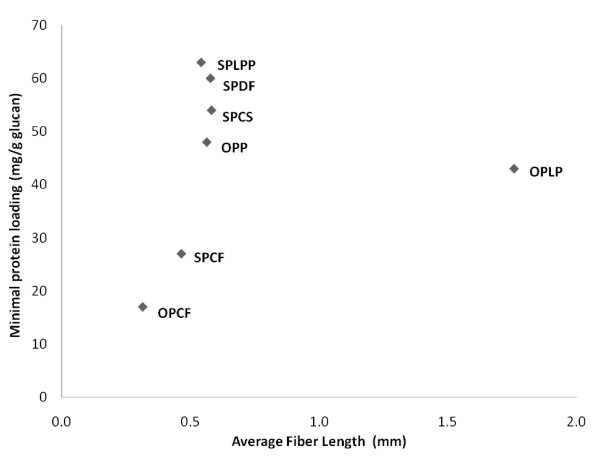
**Relationship between minimum protein loading for efficient hydrolysis and external surface area determined as average initial fiber length**.

In this study, we assessed the influence of the exterior surface area of the cellulosic-rich materials, determined by fiber dimension/length, on the minimum protein requirement for effective enzymatic digestibility of pretreated lignocellulosic substrates, using a FQA, which is an automated particle size analyzer. We found that the minimum protein requirement for efficient hydrolysis had no correlation with the average initial particle size (Figure 5). Several factors could explain this lack of correlation, including the fact that the FQA analysis provides only a gross estimation, as it assumes that the fiber particles are smooth and it does not consider the surface topology and porosity (cracks and fissures) of the particles. Additionally, the size of cellulosic particles can be difficult to measure because of the presence of different types of particles and their agglomerates [53]. Another possibility is that the minimum protein requirement for efficient hydrolysis is independent of the overall external surface area of the lignocellulosic substrate. This is not unexpected, as fiber dimensions, although a good indicator of the external surface area of cellulosic materials, do not necessarily reflect the overall cellulose surface area available to the cellulase enzymes in the pretreated lignocellulosic materials.

**Figure 6:**
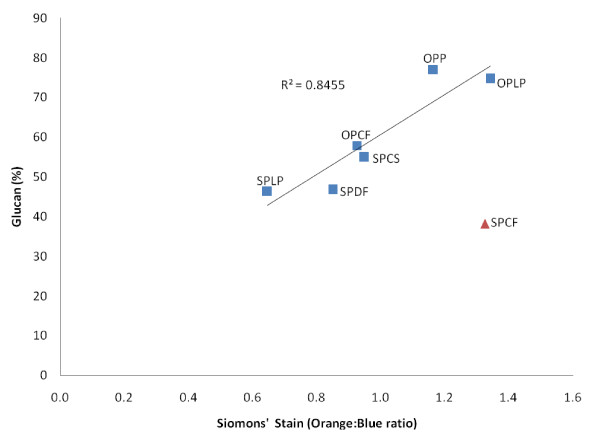
**Relationship between glucan content and distribution of large and small pores (combination interior/exterior surface area) determined by the Simons' staining technique**.

It is known that cellulose microfibrils are porous substrates, and their overall accessible surface area is expected to be a combination of exterior and interior surface area (for example, substrate porosity and topology). One method that we have adapted is the SS technique, which measures a combination of both the interior and exterior surface area of the exposed/accessible cellulose [20,35]. SS is a two-color differential stain that is sensitive to variations in the accessibility of the interior structure of fibers [54]. When the cellulosic substrates are treated with a mixture of DO and DB dyes, the DB molecules initially populate the pores of the fibers, then the DO molecules gain access to the larger pores and displace the DB molecules because of the higher molecular size and higher affinity of the DO dye [9,43]. In the present study, in addition to the exterior area estimated by measuring particle size, the ratios of adsorbed DO and DB onto the pretreated lignocellulosic materials were used to assess the overall accessible surface area of cellulose to cellulases (Figure 6). It has been shown previously that the molecular diameter of the DO dye molecules is in the range of 5 to 36 nm [54], which is close to the molecular diameter of a 'typical' fungal cellulase.

**Figure 7:**
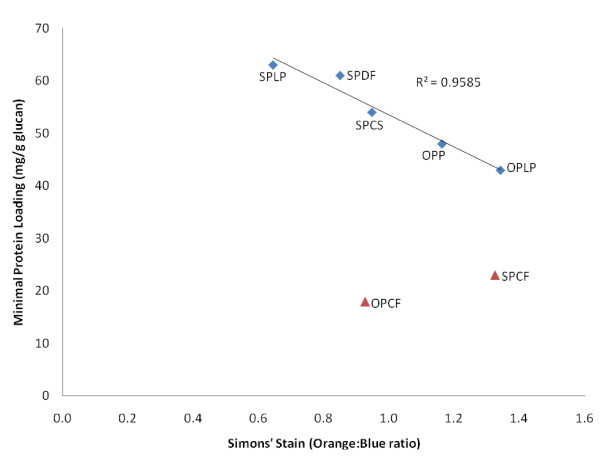
**Relationship between distribution of large and small pores (combination interior/exterior surface area) and minimum protein loading for efficient hydrolysis**.

The overall available surface area of cellulose increased with the increasing glucan content of the pretreated substrates, with the exception of SPCF, as evidenced by the linear correlation observed between the glucan content and the DO:DB ratio (Figure 6). This confirmed the previous suggestion [35] that the use of SS dyes, more specifically the DO:DB ratio, as molecular probes is a good indicator of the total (external and internal) surface area of cellulose available to the enzymes. It was also evident that the higher the DO:DB ratio, the lower the protein loading required for efficient hydrolysis (Figure 7). This indicated the strong dependency of the minimum protein requirement on the accessibility of the available cellulose in the pretreated lignocellulosic materials, and the importance of both the external and the internal surface areas (for example, pore volume, fissures and micro-cracks).

It has been reported that the internal surface area of cellulose is much larger than the external surface area [15]. Therefore, it seems logical that the porous structure of cellulose has a major influence on the diffusion of reactants such as cellulase enzymes into the cellulose network. This is in good agreement with the proposed mechanism of enzymatic hydrolysis of cellulose by cellulases, which suggests that, rather than cellulose fibrils being slowly eroded by surface 'shaving' or 'planing', the cellulase enzymes enter through pores large enough to accommodate them, facilitating the disaggregation and fragmentation of the cellulose. Therefore, the topology/porosity of the available cellulose is an important factor that may play a key role in limiting the amount of protein that can penetrate into the microfibril defects/pores of the cellulose. Previous work by Thygesen *et al*. [17] supports this suggested mechanism; they showed that cellulases first penetrated into the porous regions of cellulose, precipitating the subsequent depolymerization. It has also been shown that enzymatic degradation does not necessarily promote cleavage in the fiber axial direction, as evidenced by significant decrease in fiber length, but not fiber width [17,55].

## Conclusions

Previous work has suggested that the limited available surface area of cellulose is a key factor that necessitates the need for relatively high enzyme dosages to attain effective cellulose hydrolysis. However, the majority of these studies used highly digestible, purified cellulosic substrates such as filter paper, Solka floc or Avicel. In the present study, we used a broad range of more realistic heterogeneous, lignocellulosic feedstocks pretreated by promising technologies under more representative conditions. Regardless of significant differences in the origin, structure and chemical composition of the feedstocks and the pretreatment process used, it appears that the minimum protein loading required for efficient hydrolysis of pretreated lignocellulosic substrates has no direct relationship with only the external surface area of the cellulose-rich materials. However, protein loading did appear to be strongly influenced by the overall enzyme accessibility, as determined by the SS technique, which as well as measuring the external cellulose surface area, also takes into account the porosity/topology of the available cellulose.

A strong linear relationship between cellulose accessibility and the minimum amount of protein required to achieve effective hydrolysis was apparent, at least with the enzyme cocktail used in this study. As regards the enzymatic mechanism, these results suggest that some of the cellulase components may initially penetrate into areas of the cellulose, particularly the amorphous regions that are large enough to accommodate cellulase enzymes, disrupting/fragmenting the cellulose fibers before significant hydrolysis of cellulose takes place.

The fact that the more available/exposed cellulose in the pretreated lignocellulosic structure required lower protein levels per gram of glucan to attain high digestion rates suggests that the rate-limiting step during hydrolysis may not be the actual catalytic cleavage of the cellulose chains *per se *but rather the limited accessibility of the enzymes to the cellulose chains within the substrate matrix.

## Competing interests

The authors declare that they have no competing interests.

## Authors' contributions

VA planned and carried out the experiments, analyzed the results and wrote the paper. JNS participated in the design of the study, helped analyzing the results and contribute to the draft of the manuscript. All authors read and approved the final manuscript.
